# Consumption of Dairy Products in Relation to Type 2 Diabetes Mellitus in Chinese People: The Henan Rural Cohort Study and an Updated Meta-Analysis

**DOI:** 10.3390/nu12123827

**Published:** 2020-12-15

**Authors:** Mengying Fan, Yuqian Li, Chongjian Wang, Zhenxing Mao, Lulu Zhang, Xiu Yang, Songyang Cui, Linlin Li

**Affiliations:** 1 Department of Epidemiology and Health Statistics, College of Public Health, Zhengzhou University, Zhengzhou 450001, China; 15836150920@163.com (M.F.); Tjwcj2005@126.com (C.W.); maozhr@gmail.com (Z.M.); lulzhang@126.com (L.Z.); 15727571433@163.com (X.Y.); sycui@foxmail.com (S.C.); 2 Department of Clinical Pharmacology, School of Pharmaceutical Science, Zhengzhou University, Zhengzhou 450001, China; liyuqian0214@126.com

**Keywords:** dairy, type 2 diabetes mellitus, cross-sectional study, meta-analysis

## Abstract

Recent studies on whether dairy consumption is associated with type 2 diabetes mellitus (T2DM) have yielded inconsistent results, so we explored the relationship between dairy consumption and T2DM through a large-sample, cross-sectional study and a meta-analysis. In the meta-analysis, summary relative risks (RRs) of 23 articles were compiled with a random effects model, and a restricted cubic spline regression model was used to explore whether there is a nonlinear relationship between dairy intake and T2DM risk. This cross-sectional study used baseline data from 38,735 participants of the Henan Rural Cohort study and the association between dairy consumption and T2DM was analyzed by a logistic regression model. The meta-analysis revealed a borderline negative significant association between total dairy intake and risk of T2DM, the RR and 95% confidence interval (CI) was 0.94; (0.89, 1.00), and the risk was lowest at 270 g daily dairy intake. In the cross-sectional study, there were 3654 T2DM patients and 68.3 percent of the respondents had no dairy intake. The average intake of dairy in the total population was 12 g per day. Fully adjusted analyses suggested positive associations, with an odds ratio (OR) comparing the highest with the zero intake of 1.34 (95% CI: 1.22, 1.48) for all participants, which was unaffected by sex. Dairy intake in rural areas of Henan province is low, and we found, in the context of overall low dairy intake, that a high intake was positively associated with T2DM, which is inconsistent with the meta-analysis results suggesting that dairy has marginal protective effects against T2DM.

## 1. Introduction

The global prevalence of type 2 diabetes mellitus (T2DM) is growing, and China is seeing a similar increase [1]. According to statistics, the prevalence rate of diabetes in China was 0.90% in 1980 [2] and 9.70% in 2007 [3], compared with the latest national estimate of 10.9% in 2013, and China has the largest number of diabetics in the world, with the total number of diabetics around 114 million, nearly a quarter of the world’s patients [4]. It is worth noting that the prevalence and mortality of T2DM in rural areas of China have been on the rise in recent decades [5]. T2DM and its complications consume a large number of medical resources, affect quality of life to a great extent, and carry a serious social and economic burden [6].

A cohort study from the United States showed that dietary factors, such as dietary patterns, dietary habits, energy intake, carbohydrate, protein, and fat intake, are a daily life behavior factor that can greatly affect the prevalence of T2DM [7]. The 2017 study on disease burden in China showed that dietary factors are at the top of the list of major risk factors for chronic diseases in China [8]. Therefore, ideal dietary factors play a very important role in preventing and controlling abnormal blood glucose metabolism. Protein is a crucial part of a daily diet, and dairy is one of the main sources of high-quality protein [9]. In recent years, research on the intake of dairy and T2DM has increased. At present, it is generally believed that the total dairy intake is not associated with T2DM risk, and yogurt consumption has a significant protective effect on T2DM [10,11,12,13,14]. On the other hand, the association between T2DM and other kinds of dairy subgroups is controversial. For example, in some high-fat dairy products, the high lipid composition may offset the potential protective benefits of dairy ingredients, while the medium-chain unsaturated fatty acids in milk and other dairy products may have a positive effect on reducing the risk of T2DM [11,15]. The complexity of the dairy subgroup led to the complexity and inconsistency of the health effects of total dairy product intake. At present, there is no agreement on the association between dairy intake and the risk of T2DM.

By the end of 2019, there were around 23 cohort and cross-sectional studies on total dairy intake and T2DM [11,16,17,18,19,20,21,22,23,24,25,26,27,28,29,30,31,32,33,34,35,36,37], but most of them came from European and American countries and Australia, and only one study focused on the Chinese population. In addition, there were some inconsistencies in the findings as above. The main objective of this study was to examine the status quo of dairy intake and its association with T2DM risk in rural China through a cross-sectional study, and to compare the differences and similarities with meta-analysis data of dairy–T2DM from all over the world, so as to explore possible causes.

## 2. Materials and Methods

### 2.1. The Henan Rural Cohort Study

#### 2.1.1. Participants

Data used in this cross-sectional study came from the Henan Rural Cohort Study, and the number of participants in the baseline survey was 39,259. The study population was the rural population of Henan province, of which 60.5% were female with ages between 18 and 79. A more detailed introduction to the Henan Rural Cohort Study can be found in a previous publication [38]. The study included the following exclusion criteria: (a) absence of dietary data (*n* = 61); (b) lack of information about blood glucose and T2DM (*n* = 71); (c) patients with cancer disease or lack of relevant information (*n* = 392). The final analysis included 38,735 participants.

This survey was approved by the Zhengzhou University Life Science Ethics Committee. All participants completed informed consent forms, and the study met the ethical standards set out in the Declaration of Helsinki.

#### 2.1.2. Dietary Assessment

Dietary intake in this study was assessed using a food frequency questionnaire (FFQ) consisting of thirteen main foods based on Dietary Guidelines for Chinese Residents and the dietary characteristics of rural residents of Henan province. The reliability and validity of this FFQ have been verified and more details can be found elsewhere [39]. The food items included staple food, livestock, poultry, fish, egg, dairy, fruit, vegetable, bean, nut, pickle, cereal and animal oil. According to the questionnaire, participants were asked to report the frequency (never, day, week, month, year) and the quantity (kilograms, grams (g)) of food they consumed in the past year. According to the consumption habits of dairy products in rural China, most dairy products are milk and milk powder, a small amount of yogurt and almost no cheese; we define the total dairy products in this study as the sum of milk, yogurt and milk powder. For each main food, dietary retrospectives were assessed using a visual photo atlas that allowed participants to assess specific food intake for each food item against a standard dose. All dietary questionnaires were completed by trained and qualified investigators who followed standardized procedures for face-to-face questioning of respondents.

#### 2.1.3. Ascertainment of T2DM

We collected fasting blood glucose data of participants in laboratory tests, combined with relevant medical records, and asked participants about their diabetes disease and medication status. The diagnostic criteria of T2DM in this study are based on American Diabetes Association (ADA) diagnostic standards [40], as follows: (a) fasting blood glucose (FPG) ≥ 7.0 mmol/L, (b) a self-reported history of diabetes with current use of insulin or oral hypoglycemic agents or (c) exclusion of type 1 diabetes mellitus, gestational diabetes mellitus and diabetes due to other causes.

#### 2.1.4. Non-Dietary Covariates

The data of other covariates in this study were obtained face to face through standardized questionnaires, which included demographic characteristics, lifestyle factors, individual and family disease history, etc. The details are as follows: age, sex, education level, occupation, family income, smoking status, alcohol consumption, physical activity level, whether the individual and his/her parents and siblings suffer from coronary heart disease, hypertension, diabetes, cancer, etc. Trained staff obtained anthropometric measurements of the subjects, including height, body fat, weight, lung capacity, grip strength, waist and hip circumference, blood pressure, etc. Body mass index (BMI) can be derived from height and weight, and the formula is as follows: BMI = weight (kilograms)/height (meters)^2^. Venous blood was collected after at least 8 h of fasting and fasting blood glucose was measured. Biochemical analysis included total cholesterol (TC), triglyceride (TG), high-density lipoprotein cholesterol (HDL-C) and low-density lipoprotein cholesterol (LDL-C).

#### 2.1.5. Statistical Analysis

The continuous variables and the categorical variables are represented as mean ± SD and percentages, respectively. Analysis of variance (ANOVA) and Chi-squared tests were used to compare the basic characteristics of each group (dairy intake was 0, 0–53.68 g/day and ≥53.68 g/day). Logistic regression analysis was conducted to calculate the odds ratio (OR) for T2DM among dairy consumption groups, using the no intake group as the reference. Models were adjusted for age, gender (model 1), model 1+ smoking, alcohol, economic status, education, physical activity, total energy intake, intake of other food, high fat total, BMI, waist circumference and family history of T2DM (model 2). TC, HDL-C, LDL-C and TG were added into model 2 to detect the mediating effect of lipid metabolism markers (model 3). In accord with socio-demographic characteristics and lifestyle variables, the relationship between dairy intake and T2DM was conducted through stratified analyses. The study population was stratified into two levels based on age (<60 year, ≥60 year), BMI (<25 kg/m^2^, ≥25 kg/m^2^) and family history of diabetes (yes, no), and into three levels based on physical activity level (low, moderate, high).

All statistical analyses were performed with SPSS Statistics ver. 22.0 (IBM, Chicago, IL, USA). The test level was set at *p* < 0.05 (two-sided).

### 2.2. Meta-Analysis

#### 2.2.1. Literature Search and Study Design

We conducted a meta-analysis with prospective cohort studies and cross-sectional studies that reported research on dairy intake and T2DM by searching the CNKI, Wanfang, PubMed, Embase and Web of Science electronic databases for the period up to December 2019. The search strategy is shown in Supplementary Materials Table S1.

#### 2.2.2. Study Selection, Data Extraction and Quality Assessment

Because dairy products included milk, cheese, butter, ice cream and yogurt in the meta-analysis, which were inconsistent with the definition of dairy products in the cross-sectional study, the cross-sectional results were not included in the meta-analysis. Inclusion and exclusion criteria for this meta-analysis were as follows: a cohort study or cross-sectional study; the results included T2D; the study’s results must include OR, relative risks (RR) or hazard ratio (HR); the study must report on dairy intake; all studies had a quality score of at least 6 points as assessed by the Newcastle Ottawa Scale(NOS) [41]. The information used for reporting or analysis was extracted independently by two authors (MYF and LLL) and the whole process had strict quality control.

#### 2.2.3. Statistical Methods

For studies reporting HRs or ORs for T2DM, we assumed that the HRs and ORs were approximately RRs. A high vs. low meta-analysis (pooled RR for the highest versus the lowest categories of dairy intake), a linear dose–response meta-analysis (generalized least squares regression for each increment of 100 g of dairy intake) [42] and a nonlinear dose–response meta-analysis (two-stage restricted cubic spline analysis with three knots at the 25th, 50th and 75th percentiles) [43] were carried out in this study. The RRs and their 95% Cis were pooled by a fixed effect model [44]. Subgroup analysis was conducted by study design (cross-sectional study, cohort study), sex (men, women, men and women), age (<50 vs. ≥50 years), follow-up period (<10 vs. ≥10 years), geographic location (Europe, United States, Asia and Australia) and number of cases (<1000 vs. ≥1000). Heterogeneity was evaluated using a combination of Q test and I^2^ statistics, if I^2^ was greater than 50%, potential heterogeneity was considered; [45] we assessed publication bias by Begg–Mazumdar’s test and Egger’s tests [46].

A two-sided *p*-value was used with a level of 0.05. All data analyses were performed by using Stata 12.1 (Stata Corp, College Station, TX, USA).

## 3. Results

### 3.1. The Henan Rural Cohort Study

Table 1 shows the basic demographic characteristics of the study population. A total of 38,735 subjects were included in this study, including 3654 patients with T2DM. Remarkably, in our study population, 68.3 percent of the respondents had no dairy intake, and the median intake of total dairy in the highest group was 54 g/day, while the average intake of dairy in the total population was only 12 g per day. The higher the intake of dairy products, the younger they were, with lower levels of smoking and physical activity; at the same time, the levels of education, income and alcohol consumption tended to increase with dairy intake (*p* < 0.05 for all).

Based on the dairy intake of each group, Table 2 shows the dietary intake status and the prevalence of T2DM, the distribution of BMI and the family history of diabetes of each group. Red meat, white meat, fish, egg, fruit, bean, nut and grain consumption were higher in the group with the highest dairy intake than in the group with no dairy intake (*p* < 0.05 for all). Furthermore, participants with higher dairy consumption generally were not overweight or obese, but they were more likely to have a family history of diabetes (*p* < 0.05 for all).

In Table 3, no association was found between dairy intake and T2DM in women in the basic model (*p* for trend > 0.05). After multivariable adjustment (model 3), higher intake of dairy products was associated with higher T2DM prevalence across all participants with an OR and 95% confidence interval (CI) of 1.34 (1.22,1.48). Fully adjusted models comparing highest intake with no dairy intake groups revealed an OR of 1.44 (95% CI: 1.24, 1.68) for men and an increased OR of 1.33 (95% CI: 1.17, 1.51) for women compared to the basic model. All results of stratified analysis were consistent with the results above, showing that there is a positive association between total dairy intake and T2DM (Supplementary Materials Table S2). This study performed a sensitivity analysis to stratify the dairy consumption groups by servings per day and found that the results of the sensitivity analysis were consistent with the results of our main analysis (Supplementary Materials Table S3).

### 3.2. Meta-Analysis

Supplementary Materials Figure S1 shows the literature selection process. Briefly, we identified a total of 3589 records. Then, 790 duplicates were excluded, by skimming the titles and abstracts; the initial screening eliminated 2603 inappropriate records, and 173 did not meet the inclusion and exclusion criteria after full-text reading. Thus, the final analysis included 23 publications (Supplementary Materials Table S4); since four publications reported results by sex [24,31,34] or sub-cohort [11], we included these results as independent studies, resulting in thirty studies included in the high vs. low meta-analysis. However, among the thirty studies, seven [16,21,28,31,32,35] did not meet the inclusion criteria of dose–response meta-analysis, so twenty-three were included in the dose–response meta-analysis. Of these articles, only one was from China, eighteen were from western countries and the remaining four were published in Asia. All included studies had NOS score ≥ 6 (Supplementary Materials Table S5).

**Figure 1:**
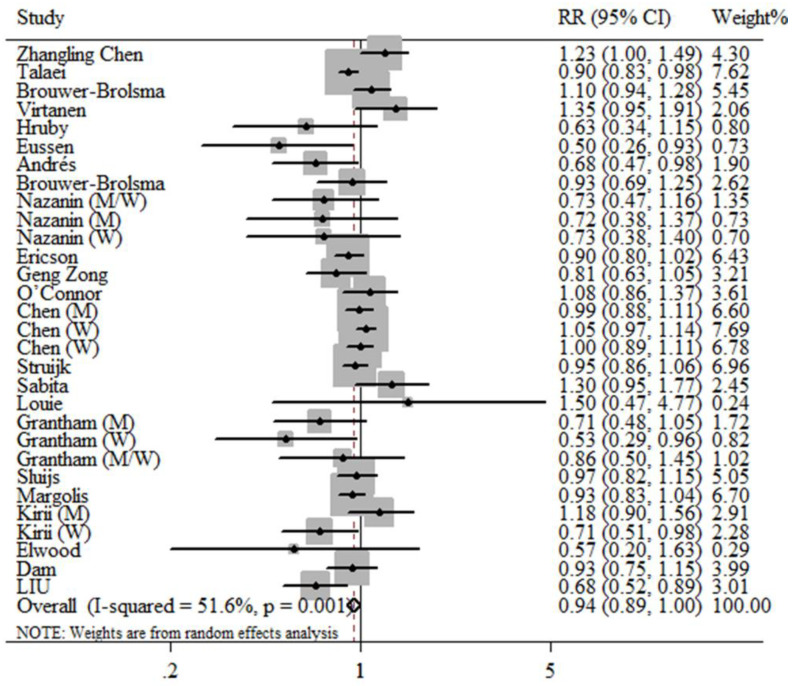
Prospective associations of high vs. low dairy intake with incident type 2 diabetes mellitus (T2DM) of the meta-analysis.

**Figure 2:**
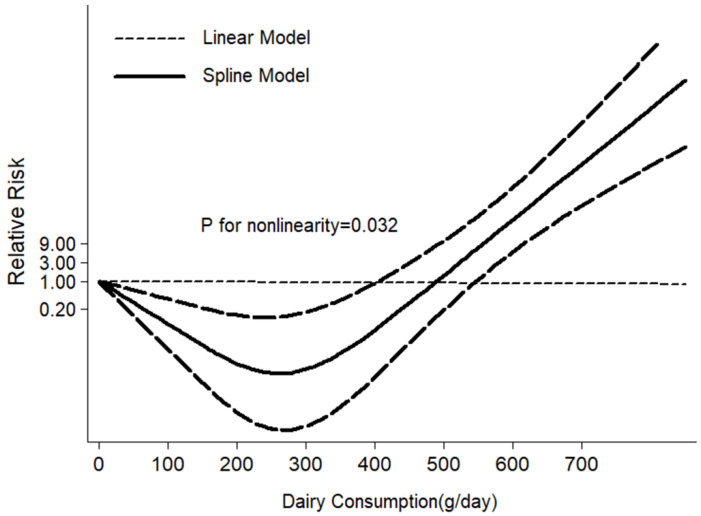
Non-linear dose–response relationship between daily intake of total dairy and T2DM risk of the meta-analysis.

The results of the high vs. low intake meta-analysis and the dose–response meta-analysis (each additional daily consumption of 100 g of dairy) both showed that dairy intake was marginally significantly associated with a decreased risk for T2DM, as shown in Figure 1 and Supplementary Materials Figure S2, and the relative risk (RRs) and 95% CI were 0.94; (0.89, 1.00), 0.98; (0.97, 1.00), respectively. The restrictive cubic spline diagram showed a nonlinear association (*p* = 0.032) between dairy intake and T2DM risk in cohort studies, and the risk of T2DM was lowest with the daily consumption of 270 g/day (Figure 2). Evidence of heterogeneity was observed between subgroups for study design, age of participants and geographic location. No association was found in the cross-sectional meta-analysis (RR: 0.79; 95% CI: 0.37, 1.69, I^2^ = 82.0%); however, a borderline negative association was found in the cohort-study meta-analysis (RR: 0.94; 95% CI: 0.89, 1.00, I^2^ = 49.0%) (Supplementary Materials Table S6). In particular, the results from studies in Asia and Australia clearly showed a protective effect of dairy intake on T2DM (RR: 0.85; 95% CI: 0.76, 0.95, I^2^ = 21.8%). Begg–Mazumdar’s test and Egger’s test, as well as the visual funnel plot, all suggested that no significant publication bias was present in our meta-analysis (Supplementary Materials Figures S3 and S4).

## 4. Discussion

Dairy intake in rural areas of Henan province is low, and we found, in the context of overall low dairy intake, that a high intake was positively associated with T2DM in the rural population of Henan, China, both in men, women and in the entire study population. It is acknowledged that this is the first study to explore the status quo of dairy intake and investigate in depth the association between dairy consumption and T2DM in the rural population in Henan, China. We also conducted a meta-analysis on dairy intake and T2DM risk, which concluded that there was a borderline positive association between dairy consumption and the risk of T2DM; the risk of T2DM was lowest when the daily consumption was 270 g/day, and in Asia and Australia, a significant protective effect of dairy intake on T2DM was found. Our study raises the possibility that differences may exist between dairy intake and T2DM among different countries, dietary patterns and ethnic groups.

Dairy products, a major source of daily protein, are rich in beneficial substances required for the physiological activities of human body, especially probiotics, unsaturated fatty acids, trans-palmitoleate, calcium and vitamin D in fermented milk, which may play a significant role in the prevention of diabetes, as indicated by several cohort studies, randomized controlled trials and animal studies [47,48,49,50]. In addition, yogurt is recognized to be beneficial to health, and it also has a preventative effect on obesity, a more reversible risk factor for cardiovascular metabolic diseases such as diabetes [51,52]. However, the complex composition of various dairy products includes some high-fat or high-sugar foods, which partly explains the complexity of the contribution of dairy products to chronic disease prevention and treatment. Less healthful substances in dairy products, such as saturated fatty acids and sugar, could weaken the role of beneficial substances, as has been demonstrated in animal studies and a population-based study [53,54]. Due to the lack of data and analysis on the association between milk, milk powder, yogurt and T2DM risk, it is not possible to obtain precise differences in the contribution of dairy subgroup variation to T2DM in our rural population in Henan province. We can only propose a hypothesis of low yogurt intake among rural people in Henan based on the surveyors’ report on the collection of dietary data. This preliminary exploratory study paves the way for further exploration of the association between dairy subgroups and diabetes.

An important point which must be emphasized is the low intake of dairy in this study region. Dairy consumption in China is much lower than that in America due to dietary patterns, living environment and ethnic differences [55]. A review of Chinese residents suggested that the average consumption of dairy was 24.7 g/day in 2012, almost twice the average intake in rural areas of Henan. This review showed that there are significant differences in dairy intake between urban and rural residents [56]. An evidence-based review suggested that cumulative average consumption of total dairy was 363 g/day and the dose–response relationship was analyzed in units of 200 g/day; this study concluded that 300–400 g/day of dairy intake was the optimal dose for T2DM prevention [57]. The group with the highest dairy intake in our study reported only 54 g/day, far below the recommended daily intake of 300 g/day in the 2016 dietary guidelines for Chinese residents [58].

Compared to the basic model, after further adjusting the other variables in the final model, the positive correlation between dairy intake and T2DM was strengthened in men and total participants, and a trend towards a positive association with T2DM and dairy intake was observed for women. These results are consistent with a 2019 cohort study from the Netherlands [16], which suggested that each 5% increment in energy from dairy was associated with a 23% increase in the risk of T2DM. A meta-analysis in 2019 also reported that high dairy intake is associated with an increased risk of T2DM (RR: 1.24; 95% CI: 1.04–1.47) [59]. However, previous research indicated that there is no significant association between dairy intake and T2DM risk [18,19,23]. Moreover, it cannot be ignored that the protective effect of total dairy intake on T2DM has also been demonstrated in a few studies. For example, a cross-sectional analysis of the Maastricht study from the Netherlands in 2016 showed that T2DM risk decreased with increasing consumption of dairy products [21]. In contrast, our study was carried out in a rural population of Henan Province, whose largely rural population has a relatively low economic status. Local residents mainly eat rice, noodles and vegetables, and dairy products are an expensive luxury food, leading to low consumption of dairy. In this study, people with relatively high dairy intake were more likely to have higher family incomes, be physically inactive and have a family history of diabetes. It should be recognized that some patients may have changed their dietary patterns and increased their dairy intake upon learning that they had diabetes.

This study not only explored the association between dairy intake and T2DM risk but also conducted an updated meta-analysis. A comprehensive analysis of a total of 23 articles was conducted and found that total dairy intake was marginally significantly associated with a decreased risk for T2DM, a conclusion reached in the results reported in the cohort studies analysis in the stratified analysis. Moreover, previous meta-analyses on the protective effect of dairy intake on T2DM have provided similar evidence [13,14,57]. It is worth exploring that there are obvious regional differences in this review. In the Asian studies, dairy products had a clear protective effect against T2DM, but this was not found in the European and U.S. studies. It is a pity that only one study was from China [26] in our meta-analysis, which suggested that there was no association between dairy intake and T2DM risk. This is not consistent with our results of the Henan Rural Cohort Study, also from the Chinese population. The economic development level, dietary patterns and racial differences in non-Chinese studies may lead to different results between this meta-analysis and the original cross-sectional analysis. In addition, the two studies from China arrived at different conclusions. This further suggests the necessity and urgency of exploring the association between dairy intake and T2DM in Asian populations, especially in China, so as to obtain more comprehensive and diversified research results and to provide scientific and reliable recommendations for the diet and health of the entire human population.

This study is the first study to explore the status quo of dairy intake and to investigate the association between dairy intake and T2DM risk in rural China. Secondly, this study is based on the Henan Rural Cohort Study, which has a large sample size, rigorous design, good quality control and comprehensive collection of information, so it is representative and reliable. Inevitably, there are still some limitations in this study; the cross-sectional study design prevented us from determining the causal association between dairy intake and T2DM and we did not conduct a dairy subgroup analysis. While we know that dairy intake and T2DM are positively related, we cannot conclude that dairy consumption is a risk factor for T2DM. One possibility is that patients who learned they had diabetes changed their diet and increased their milk intake, so it is necessary to add a time factor and a factor of dairy composition variability to the cohort study analysis to obtain more accurate results that reflect causality. In future studies, we will use follow-up data to examine whether diabetic patients may change their eating habits and increase their dairy intake due to disease. Another point is that the dietary data were obtained by participants recalling their dietary intake over the past year. Therefore, there may be some recall bias, leading to some errors in the results. To test whether retrospective lag between the timing of ingestion and data collection would make the data unreliable, previous study by our team [39] conducted reliability and validity testing on the FFQ through a 24-h dietary review method over 3 days, which indicated that the dietary data were authentic and reliable. The last point is that our research study is restricted to Henan province and may have some limitations when it is extended to the whole country.

## 5. Conclusions

In conclusion, the consumption of total dairy products in rural Henan province is far lower than that recommended by Chinese dietary guidelines. The fact that nearly 70 percent of people have not consumed dairy in the past year also reflects the lack of nutrition in rural areas of Henan province. We found that high dairy intake is positively associated with T2DM within the low dairy intake environment of the study population, but this was inconsistent with the results of the meta-analysis, where total dairy intake was borderline significantly associated with a decreased risk of T2DM. These discrepant findings suggest that cohort studies and randomized clinical trials are needed to further investigate the cause and effect of dairy intake on T2DM risk. The results of this cross-sectional study also point out the direction for future studies on the relationship between dairy and T2DM in rural China.

## Figures and Tables

**Table 1:** General characteristics of participants (*n* = 38,735 in total) of the Henan Rural Cohort Study according to total intake of dairy products.

	Intake (g/day) of Dairy Products			
	0	0–53.68	≥53.68	*p*-Value ^a^
No. subjects	26,466 (68.33)	5922 (15.29)	6347 (16.62)	
Male sex	10,382 (39.23)	2456 (41.47)	2496 (39.33)	0.005
Age (year)	56.37 ± 11.12	54.20 ± 13.01	53.49 ± 15.04	<0.01
Smoking status				0.002
Never	19,262 (72.78)	4228 (72.41)	4675 (72.63)	
Ever	2068 (7.81)	538 (9.08)	525 (8.16)	
Current	5136 (19.41)	1156 (18.51)	1147 (19.21)	
Drinking status				<0.01
Never	20,667 (78.09)	4392 (74.16)	4855 (76.49)	
Ever	1136 (4.29)	298 (5.03)	359 (5.66)	
Current	4663 (17.62)	1232 (20.80)	1133 (17.85)	
Physical activity				<0.01
Low	8131 (30.72)	1906 (32.19)	2466 (38.85)	
Moderate	9997 (37.77)	2303 (38.89)	2314 (36.46)	
High	8338 (31.50)	1713 (28.93)	1567 (24.69)	
Education level				<0.01
Illiterate	4891 (18.48)	820 (13.85)	738 (11.63)	
Primary school	7977 (30.14)	1491 (25.18)	1420 (22.37)	
Middle school	10,422 (39.38)	2514 (42.45)	2502 (39.42)	
High school	2767 (10.45)	863 (14.57)	1191 (18.76)	
University or higher	409 (1.55)	234 (3.95)	496 (7.81)	
Per capita monthly income				<0.01
<500 RMB	9978 (37.70)	1941 (32.78)	1891 (29.79)	
500–999 RMB	8851 (33.44)	1967 (33.22)	1938 (30.53)	
1000–1999 RMB	6041 (22.83)	1517 (25.62)	1730 (27.26)	
2000–2999 RMB	1094 (4.13)	304 (5.13)	497 (7.83)	
≥3000 RMB	502 (1.90)	193 (3.26)	291 (4.58)	

Grouped according to daily dairy intake (g/day): 0; 0–53.68; ≥53.68; Data are presented as the mean ± SD or as *n* (%); ^a^ *p*-values were determined using χ2 tests for categorical variables, analysis of covariance (ANCOVA) for continuous variables; RMB: Renminbi.

**Table 2:** Dietary intake and type 2 diabetes mellitus (T2DM) characteristics of participants according to the dairy intake of the Henan Rural Cohort Study.

Variable	Total Dairy (g/day) ^a^			
	0	0–53.68	≥53.68	*p*-value ^b^
Staple food (g/day) ^c^	425.19 ± 152.93	432.82 ± 160.32	405.95 ± 155.60	<0.001
Livestock (g/day) ^d^	14.29 (5–35.71)	16.67 (7.14–42.86)	21.43 (8.33–50.00)	<0.001
Poultry (g/day) ^d^	6.67 (1.37–16.67)	7.14 (3.33–16.67)	8.33 (3.33–28.57)	<0.001
Fish (g/day) ^d^	0.82 (0–3.33)	1.67 (0.27–5.48)	2.74 (0.27–8.33)	<0.001
Egg (g/day) ^d^	52.60 ± 44.82	55.29 ± 43.51	67.64 ± 44.91	<0.001
Fruit (g/day) ^d^	129.23 ± 132.68	152.84 ± 141.33	175.32 ± 144.07	<0.001
Vegetable (g/day) ^d^	316.93 ± 179.21	320.80 ± 182.12	318.60 ± 184.41	0.388
Bean (g/day) ^d^	14.29 (3.33–35.71)	16.67 (7.14–42.86)	28.57 (10.96–57.14)	<0.001
Nut (g/day) ^d^	5.48 (0–16.67)	7.14 (1.67–21.43)	8.33 (1.67–33.33)	<0.001
Cereal (g/day) ^d^	35.00 (10.96–85.71)	33.33 (13.33–71.43)	50.00 (16.67–100.00)	<0.001
BMI (kg/m^2^) ^e^				<0.001
Underweight	560 (2.12)	162 (2.74)	215 (3.39)	
Normal	10,209 (38.57)	2503 (42.27)	2814 (44.34)	
Overweight	10,612 (40.10)	2294 (38.74)	2370 (37.34)	
Obesity	4998 (18.88)	947 (15.99)	923 (14.54)	
T2DM ^e^	2502 (9.45)	481 (8.12)	671 (10.57)	<0.001
Family history of diabetes ^e^	1029 (3.89)	282 (4.76)	303 (4.77)	<0.001

^a^ Cut-offs are based on absolute intakes; ^b^ *p*-value as calculated using analysis of variance (ANOVA) or Kruskal–Wallis ANOVA for non-parametric data; ^c^ Mean ± SD; all values in row; ^d^ Median (interquartile range); all values in row; ^e^ *n* (%); all values in row. BMI, body mass index.

**Table 3:** ORs of T2DM according to the intake of dairy products (*n* = 38,735) of the Henan Rural Cohort Study.

	Intake (g/day) of Dairy Products			
	0	0–53.68	≥53.68	*p*-Value for Trend
All Cases/*n*	2502/26,466	481/5922	671/6347	
Model 1	1.00	0.90 (0.81,0.99)	1.20 (1.10,1.32)	<0.01
Model 2	1.00	0.96 (0.86,1.07)	1.31 (1.19,1.45)	<0.01
Model 3	1.00	0.97 (0.87,1.08)	1.34 (1.21,1.48)	<0.01
Men Cases/*n*	905/10,382	194/2456	290/2496	
Model 1	1.00	0.92 (0.78,1.08)	1.38 (1.20,1.59)	<0.01
Model 2	1.00	0.98 (0.83,1.16)	1.41 (1.21,1.64)	<0.01
Model 3	1.00	0.97 (0.82,1.15)	1.44 (1.24,1.68)	<0.01
Women Cases/*n*	1597/16,084	287/3466	381/3851	
Model 1	1.00	0.90 (0.79,1.03)	1.12 (0.99,1.26)	0.16
Model 2	1.00	0.97 (0.85,1.12)	1.30 (1.14,1.47)	<0.01
Model 3	1.00	0.99 (0.86,1.14)	1.33 (1.17,1.51)	<0.01

odds ratio, OR. Model 1: adjusted for age, sex. Model 2: Model 1 plus smoking, alcohol, physical activity, per capita monthly income, education level, energy, staple-food, livestock, poultry, fish, egg, fruit, vegetables, bean, nut, grains, high fat total, BMI, waist circumference, family history of T2DM. Model 3: Model 2 plus total cholesterol (TC), triglyceride (TG), high-density lipoprotein cholesterol (HDL-C), low-density lipoprotein cholesterol (LDL-C).

## Supplementary Materials

The following are available online at https://www.mdpi.com/2072-6643/12/12/3827/s1, Table S1: Search terms in meta-analysis of association between total dairy and type 2 diabetes mellitus; Table S2: Type 2 diabetes mellitus according to total dairy intake: stratified analyses of the Henan Rural Cohort Study; Table S3. ORs of T2DM according to servings per day of dairy products (*n* = 38,735) of the Henan Rural Cohort Study; Table S4: Characteristics of included studies of total dairy consumption and type 2 diabetes mellitus of the meta-analysis; Table S5: Quality assessment of cohort studies included in meta-analysis (Newcastle–Ottawa Quality Assessment Scale); Table S6: Dose–response meta-analysis for per 100 g/day increase in total dairy and risk of type 2 diabetes mellitus, stratified by study design, gender, age, follow-up, geographic location, number of cases of high vs. low intake; Figure S1: Flow diagram of the meta-analysis; Figure S2. Prospective associations of dairy intake with incident type 2 diabetes mellitus for dose–response analysis of the meta-analysis; Figure S3: Funnel plots of dairy intake–T2D associations (high vs. low meta-analysis) SE = Standard error; Figure S4: Funnel plots of dairy intake–T2D associations (dose–response meta-analysis) SE = Standard error.

## Abbreviations

T2DM	type 2 diabetes mellitus
FFQ	food frequency questionnaire
ADA	American Diabetes Association
FPG	fasting blood glucose
BMI	body mass index
TC	total cholesterol
TG	triglyceride
HDL-C	high-density lipoprotein cholesterol
LDL-C	low-density lipoprotein cholesterol
NOS	Newcastle Ottawa Scale
ANOVA	analysis of variance
OR	odds ratio
CI	confidence intervals
RR	relative risk
HR	hazard ratio
