# Supplementary material for: Consumption of Dairy Products in Relation to Type 2 Diabetes Mellitus in Chinese People: The Henan Rural Cohort Study and an Updated Meta-Analysis
Source: Nutrients. 2020 Dec 15;12(12):3827. doi: 10.3390/nu12123827 (PMC7765212; doi:10.3390/nu12123827)
Supplement: Supplementary file 1 [file nutrients-12-03827-s001.pdf]

Supplementary table 1 Search terms in meta-analysis of association between total dairy and type 2 diabetes mellitus

Search date	Search term
2019.12.0.2	#1 milk*[tiab] OR dairy*[tiab] OR dairy product*[tiab] OR cheese*[tiab] OR butter*[tiab] OR cream*[tiab] OR yogurt*[tiab]<br>#2 diabetes<br>#3 prospective OR follow-up OR cohort OR longitudinal OR cross sectional<br>#4 (#1 AND #2 AND #3)

Supplemental Table 2 Type 2 diabetes mellitus according to total dairy intake: stratified analyses of the Henan Rural Cohort Study

	Median intake （g/d） of dairy products			
	0	16.67	53.68	*p* value for trend[a]
Stratified by age				
<60y (1520 cases)	1.00	0.95 (0.81,1.12)	1.31 (1.11,1.54)	0.002
≥60y (2134 cases)	1.00	1.07 (0.93,1.24)	1.56 (1.38,1.77)	<0.0001
Stratified by BMI				
<25 kg/m^2 (1409 cases)	1.00	1.03 (0.87,1.21)	1.38 (1.19,1.60)	<0.0001
≥25 kg/m^2 (2245 cases)	1.00	0.94 (0.81,1.08)	1.31 (1.15,1.49)	<0.0001
Stratified by physical activity level				
Low (1432 cases)	1.00	1.11 (0.93,1.32)	1.37 (1.18,1.60)	<0.0001
Moderate (1291)	1.00	0.87 (0.73,1.04)	1.28 (1.09,1.52)	0.009
High (931 cases)	1.00	0.90 (0.73,1.12)	1.34 (1.10,1.64)	0.009
Stratified by diabetes family history				
Yes (361 cases)	1.00	0.75 (0.51,1.10)	1.62 (1.14,2.32)	0.023
No (3293 cases)	1.00	1.00 (0.89,1.12)	1.32 (1.19,1,47)	<0.0001

[a]*P*-trend was calculated by assigning median values to each group and was treated as continuous variable.

[b]All multivariate models were adjusted for age, sex, smoking, alcohol, physical activity, per capita monthly income, education level, energy, staple-food, livestock, poultry, fish, egg, fruit, vegetables, bean, nut, grains, HF-total, BMI, waist circumference, family history of T2DM, TC, HDL-C, TG, LDL-C.

Supplementary Table 3. ORs of T2DM according to servings per day of dairy products(n=38735) of the Henan Rural Cohort Study.

	Servings per day of dairy products			
	**0**	**0-1.00**	**≥1.00**	***p* value for trend**
All				
Cases/*n*	2502/ 26466	475/ 5828	677/ 6441	
Model 1	1.00	0.90 (0.81,1.00)	1.20 (1.09,1.31)	<0.01
Model 2	1.00	0.96 (0.86,1.07)	1.29 (1.17,1.42)	<0.01
Model 3	1.00	0.97 (0.87,1.08)	1.34 (1.21,1.48)	<0.01
Men				
Cases/*n*	905/10382	192/2425	292/2527	
Model 1	1.00	0.92 (0.78,1.08)	1.38 (1.20,1.58)	<0.01
Model 2	1.00	0.98 (0.82,1.16)	1.40 (1.21,1.63)	<0.01
Model 3	1.00	0.97 (0.82,1.15)	1.43 (1.20,1.66)	<0.01
Women				
Cases/n	1597/16084	283/3403	385/3914	
Model 1	1.00	0.90 (0.79,1.03)	1.11 (0.98,1.25)	0.65
Model 2	1.00	0.97 (0.85,1.12)	1.29 (1.14,1.46)	<0.01
Model 3	1.00	0.99 (0.86,1.14)	1.32 (1.16,1.50)	<0.01

1 serving = 50g; Model 1: adjusted for age, sex. Model 2: Model 1 plus smoking, alcohol, physical activity, per capita monthly income, education level, energy, staple-food, livestock, poultry, fish, egg, fruit, vegetables, bean, nut, grains, high fat-total, BMI, waist circumference, family history of T2DM. Model 3: Model 2 plus TC, HDL-C, TG, LDL-C.

Supplementary table 4 Characteristics of included studies of total dairy consumption and type 2 diabetes mellitus of the meta-analysis

**First Author**	**Publication year**	**Study name**	**Country**	**Follow-up time (year)**	**Population**	**Method of dietary exposure assessment**	**Method of diabetes assessment**	**Adjustment variables**
Chen	2019	RS-I RS-II RS-III	Netherlands	7.2	≥45; 41.4%men; 643 cases, 6813 subjects	170-item FFQ, 389-item FFQ;	1.Fasting blood glucose concentration of 7.0mmol/L or higher 2.Non- fasting blood glucose concentration of 11.1mmol/L or higher 3. The use of hypo-glycemic drugs	Total fat intake, total energy intake, alcohol intake, age, sex, smoking status, education level, diet quality score, physical activity and family history of diabetes, time point of WC measurements, longitudinal WC, the interaction between protein intake and longitudinal WC
Talaei	2018	SCHS	China	12	45–74; 42.7 %men; 5207 cases, 45426 subjects	165-item semi-quantitative FFQ;	Self-reported	Age, sex, dialect, year of interview, educational level, body mass index, physical activity, smoking status, alcohol use, baseline history of self-reported hypertension, total energy intake, vegetable, fruit, soy-rich pattern, dim sum, meat-rich pattern, coffee, soda

Brouwer-Brolsma	2018	the Lifelines Cohort Study	Netherlands	0	45 ± 13; 41%men; 1305 cases; 112086 subjects	flower FFQ	ND-T2DM was defined as having a FPG ⩾7•0 mmol/L or HbA1c⩾6•5%	age, sex , alcohol, smoking , education , physical activity , total energy intake , the intake of energy-adjusted bread, pasta, rice, potato, fruit, vegetables, legumes, meat, fish, coffee, tea, soda/fruit juice, other dairy product groups , BMI and waist circumference
Virtanen	2017	KIHD	Finland	19.3	53.1±5.2; 100%men; 432 cases; 2332 subjects	4-d dietary records;	Self-administered questionnaires, fasting blood glucose measurements, 2-h oral glucose tolerance tests, national registers	Age, examination year, energy intake, marital status, income, use of hypertension medication, family history of diabetes, pack-years of smoking, education, leisure-time physical activity, serum ferritin, alcohol intake, glycaemic index, and dietary intakes of fibre, Mg, coffee, cholesterol, and SFA, MUFA, PUFA and trans-fatty acids, BMI, fasting plasma glucose and fasting serum insulin
Hruby	2017	The National Heart, Lung, and Blood Institute s Framingham Heart Study Offspring Cohort	USA	12	54.0±9.7; 46% men; 196 cases; 2809 subjects	126-item FFQ	use of an oral hypoglycemic drug or insulin, or the first measurement of FG ⩾7.0 mmol/L (⩾126 mg/dL)incident	age, sex, energy intake, parental history of diabetes, baseline smoking status, dyslipidemia or treatment, hypertension or treatment, means of other dietary characteristics, including intake of coffee, nuts, fruits, vegetables, meats, alcohol, and fish; the glycemic index, other dairy intake, baseline BMI, weight change over follow-up
Simone	2016	the Maastricht Study	Netherlands	0	59.9±8.2; 50.9% men; 125 cases; 2391 subjects	the Dutch national FFQ tool	Oral glucose tolerance test	age ,sex, BMI, physical activity, smoking status, education and intakes of energy, vegetables, fruits, meat and fish

Brouwer-Brolsma	2016	RS	Netherlands	9.5	65.1±6.7; 40%men; 393 cases; 2974 subjects	170 food items FFQ;	Records of general practitioners' (including laboratory glucose measurements), hospital discharge letters, and serum glucose measurements	Age, sex, alcohol , smoking, education, physical activity , BMI, total energy intake, energy adjusted meat intake, energy-adjusted fish intake, potential intermediates
Andrés	2016	PREDIMED	Spain	4.1	66.6±6.6; 38.4% men; 270 cases; 3454 subjects	137-item semi-quantitative FFQ;	Clinical diagnosis or use of anti diabetic medication	Age, sex, BMI, dietary intervention group, leisure time physical activity, educational level , smoking, hypertension or antihypertensive use ,fasting glucose, HDL-cholesterol, triglyceride concentrations, cumulative average consumption of dietary variables in energy-adjusted quintiles, alcohol
Nazanin	2015	TLGS	Iran	9	43.6±12.0; 45.7% men; 178 cases; 520 subjects	168-item semi-quantitative food frequency questionnaire (FFQ)	the American Diabetes Association (ADA) at follow-up examinations: (1) fasting plasma glucose (FPG) 126 mg/dL (7 mmol/L) or (2) 2 h-plasma glucose (2 h-PG) after consumption of 75 g glucose (OGTT) 200 mg/dL (11.1 mmol/L)	age, sex, date of blood drawn, controlled for family history of diabetes, BMI at baseline, WCBMI at baseline, total energy intake, high BP, high TG , high cholesterol at baseline, BMI change
Ericson	2015	MDC	Sweden	14	45–74; 38.8%men; 2860 cases; 24070 subjects	A 7-d menu book, a 168-item questionnaire, a 45-min interview;	Used information on the date of diagnosis from the registers prioritised in the following order: (1) the Regional Diabetes 2000 Register of Scania (2) the Malmo HbA1c Register (3)the Swedish National Diabetes Register	Age, sex, method version, season, education, BMI, leisure-time physical activity, smoking, intakes of total energy and alcohol
O'Connor	2014	EPIC-Norfolk Study	UK	11	40–79; 44%men; 752 cases; 4126 subjects	130-item semi-quantitative FFQ,7-d food diary;	Self-report confirmed by record linkage with several databases	Age, sex, BMI, family history of diabetes, smoking, alcohol, physical activity, social class, education level, energy, fiber, fruit, vegetables, red

								meat, processed meat, coffee intake
Geng	2014	NHAPCS	China	6	50–70; 41%men; 507 cases; 2091 subjects	74-item FFQ;	Self-report, Use of any oral hypoglycemic medication or insulin, or fasting glucose ⩾7.0 mmol/L	Age, sex, region, residence, smoking, family history of diabetes, BMI, dietary fiber intake, changes in BMI and waistline
Chen	2014	NHS	USA	30	52±9.3; Women; 7841 cases; 76874 subjects	61-item FFQ; 131-item FFQ	biennial questionnaire were mailed a supplementary questionnaire about symptoms and treatment	age, alcohol consumption , physical activity level, smoking status , race , menopausal status and hormone use in women, family history of diabetes, history of hypertension and hypercholesterolemia, quintiles of total calories, and dietary score, a BMI category
		NHS II		16	46±7.1; Women; 3951 cases; 88262 subjects	131-item FFQ		
		HPFS		24	36±4.6 Men; 3364 cases; 41349 subjects	131-item FFQ		
Sabita	2013	Whitehall II study	UK	10	56±6.1; 72%men; 273 cases; 4186 subjects	Validated 114 items FFQ;	Self-report of doctor's diagnosis, initiation of anti diabetic medication, a 2-h 75-g oral-glucose tolerance test	Age, sex, ethnicity, employment grade, smoking, alcohol intake, BMI, physical activity, family history of coronary heart disease/hypertension , fruit, vegetables, bread, meat, fish, coffee, tea, total energy intake
Struijk	2013	Inter99 Study	Denmark	5	30-60; 48%men; 214 cases; 5232 subjects;	Validated 114 items FFQ;	Fasting plasma glucose ⩾7.0 mmol/L and/or 2-h plasma glucose ⩾11.1 mmol/L based on 1oral-glucosetolerance test	Age, sex, intervention group, diabetes family history, education level, physical activity, smoking status, intake of alcohol, wholegrain cereal, meat, fish, coffee, tea, fruit, vegetables, energy, change in diet form baseline to 5-y follow-up, waist circumference
Louie	2013	the Blue Mountains Eye Study (BMES)	Australia	10	63±8.1; 44% men; 145 cases; 1824 subjects	a 145-item self administered semi-quantitative FFQ	Fasting blood glucose > 5.6 mmol/L or diagnosed T2DM	age, sex systolic blood pressure , baseline BMI status, HDL cholesterol , total cholesterol ,triglyceride , calcium

Grantham	2013	AusDiab	Australia	5	25-88; 45%men; 209 cases; 5582 subjects	121-itemFFQ	Fasting plasma glucose ≥7.0 mmol/L or 2-h post load plasma glucose ≥11.1 mmol/L or treatment with insulin or oral hypoglycemic agents	Age, sex, energy intake, family history of diabetes, education level, level of physical activity, smoking status, triglycerides, HDL cholesterol, systolic blood pressure, waist circumference, hip circumference
Sluijs	2012	(EPIC)-Inter Act case-cohort study	Europe	12.3	52.2±8.9; 38%men; 12403cases ; 340234 subjects	self or interviewer-administered dietary questionnaires	using multiple sources of evidence including self report, linkage to primary care registers and secondary care registers, medication use (drug registers), hospital admissions, and mortality data	center, age, sex, BMI, educational level, smoking status, physical activity level, and alcohol intake, intake of energy and energy-adjusted intakes of fruit plus vegetables, red meat, processed meat, sugar sweetened soft drinks, coffee, cereals, cereal products, dietary intake of calcium, magnesium, and vitamin D
Margolis	2011	WHI-OS	USA	8	50–79; Women; 3946 cases; 82073 subjects	a semiquantitative FFQ designed for the WHI that inquired about the average use of 122 line items	self-reports of diabetes treatment were matched by the medication inventory	age, race/ethnicity, total energy intake, income, education, smoking, alcohol intake, family history of diabetes, use of postmenopausal hormone therapy, systolic blood pressure, diastolic blood pressure, BMI, and physical activity, dietary glycemic load , dietary total fat , dietary total fiber, total magnesium
Kirii	2009	JPHC	Japan	5	45-75; 42.9% men; 1114 cases; 59796 subjects	147-item FFQ;	Self-administered questionnaire	Age , area , BMI, family history of diabetes mellitus, smoking status , alcohol intake, history of hypertension, exercise frequency, consumption of coffee , energy-adjusted magnesium, total energy

Elwood	2007	Caerphilly	UK	25	45-59; Men; 41 cases; 640 subjects	a 7-day weighed dietary intake record	Plasma insulin level ≥163mmol/l and/or plasma glucose ≥6.1mmol/l	Age, smoking, BMI, social class
Dam	2006	Black Women's	USA	8	21–69; Women; 1964 cases; 41186 subjects	68-item baseline Block food frequency questionnaire	Fasting plasma glucose ≥7.0mmol/L or 2h post-load plasma glucose ≥11.1mmol/L	Age, energy, BMI, smoking, physical activity, alcohol consumption, parental history of diabetes, education, coffee, diet.
Liu	2006	WHS	USA	10	≥ 45; 0% men; 1603 cases; 39876 subjects	A validated SFFQ that inquired about the average use of 131 foods and beverages;	By asking women to report these items on annual follow-up Questionnaires; validated by using the ADA criteria; primarily obtain additional information with a telephone interview and supplemental questionnaire	Total energy intake, randomized-treatment assignment, age, family history of diabetes, smoking status, BMI, hypercholesterolemia, hypertension, physical activity, hormones, alcohol consumption, dietary intakes, fibers, total fat, dietary glycemic load, quintiles of dietary, calcium, vitamin D, magnesium.

Supplementary table 5 Quality assessment of cohort studies included in meta-analysis (Newcastle-Ottawa Quality Assessment Scale)

First author, year	Cohort	Selection					Comparability			Outcome				Total (9Max)
		Q1	Q2	Q3	Q4	Sub-Total	Q1 A*	Q1 B**	Sub-Total	Q1	Q2	Q3	Sub-Total	
Chen 2019	RS-I	1	1	1	1	4	1	1	2	1	1	0	2	8
	RS-II	1	1	1	1	4	1	1	2	1	1	0	2	8
	RS-III	1	1	1	1	4	1	0	1	1	1	0	2	7
Talaei 2018	SCHS	1	1	1	1	4	1	0	1	0	1	1	2	7
Brouwer-Brolsma1 2018	the Lifelines Cohort Study	1	1	1	0	3	0	1	1	1	1	0	2	6
Virtanen 2017	KIHD	1	1	1	1	4	1	1	2	1	1	1	3	9
Hruby 2017	The NHL and BFHSOC	0	1	1	1	3	1	0	1	1	1	1	3	7
Eussen 2016	the Maastricht Study	0	1	1	1	3	0	1	1	0	1	1	2	6
BrouwerBrolsma 2016	the Rotterdam Study	1	1	1	1	4	1	0	1	1	1	1	3	8
Andrés 2016	PREDIMED	0	1	1	1	3	1	0	1	1	0	1	2	6
Moslehi 2015	TLGS	1	1	1	1	4	0	1	1	0	1	1	2	7
O'Connor 2014	EPIC-Norfolk Study	1	1	1	1	4	1	1	2	0	1	1	2	8
Geng 2014	NHAPC	1	1	1	1	4	0	0	0	0	1	1	2	6
Sabita 2013	Whitehall II study	1	1	1	1	4	1	0	1	1	1	1	3	8
Struijk 2013	Inter99 Study	1	1	1	1	4	1	1	2	1	1	0	2	8
Kirii 2013	JPHC	1	1	1	1	4	1	1	2	0	1	0	1	7
Ericson 2015	MDC	1	1	1	1	4	1	0	1	1	1	0	2	7
Louie 2013	BMES	1	1	1	1	4	0	1	1	1	0	0	1	6
Grantham 2013	AusDiab	1	1	1	1	4	1	1	2	1	0	0	1	7
Sluijs 2012	EPIC)-InterAct case-cohort study	1	1	1	1	4	1	0	1	1	0	1	2	7
Chen 2014	NHS	0	1	1	1	3	1	1	2	0	1	1	2	7
	NHS II	0	1	1	1	3	1	1	2	0	1	1	2	7
	HPFS	0	1	1	1	3	1	1	2	0	1	1	2	7
Karen 2011	WHI-OS	1	1	1	1	4	1	0	1	1	1	1	3	8
Liu 2006	WHS	1	1	1	1	4	1	1	2	0	1	1	2	8
Elwood 2007	CAPS	1	1	1	1	4	1	0	1	0	1	0	1	6
Dam 2006	BWHS	1	1	1	1	4	1	1	2	0	1	1	2	7

*adjusted for age and sex

** adjusted for age, sex, BMI, energy intake, physical activity, smoking, alcohol, family history of diabetes

Supplementary Table 6 Dose-response meta-analysis for per 100g/day increase in total dairy and risk of type 2 diabetes mellitus, stratified by study design, gender, age, follow-up, geographic location, number of cases of high vs. low intake

Dietary factor	No of studies	RR	95% CI	I^2 (%)	P
Total dairy					
High vs. low	30	0.94	0.89, 1.00	51.6	0.001
Dose-response	23	0.98	0.97, 1.00	55.2	0.001
Study design					
Cross-sectional study	2	0.79	0.37, 1.69	82.0	0.018
Cohort study	28	0.94	0.89, 1.00	49.0	0.002
Gender					
Women	7	0.97	0.84, 1.12	55.9	0.034
Men	15	0.95	0.86, 1.04	53.2	0.008
Men and women	8	0.92	0.83, 1.05	55.7	0.027
Age					
<50	8	1.00	0.95, 1.06	12.1	0.336
≥50	22	0.92	0.85, 1.00	55.2	0.001
Follow-up					
<10 years	16	0.90	0.81, 0.99	54.0	0.005
≥10 years	14	0.97	0.91, 1.04	51.1	0.014
Geographic location					
Europe	10	1.00	0.88, 1.13	63.9	0.003
America	11	0.97	0.91, 1.04	41.9	0.088
Australia/Asia	9	0.85	0.76, 0.95	21.8	0.235
Number of cases					
<1000	21	0.92	0.82, 1.02	51.6	0.003
≥1000	9	0.96	0.90, 1.02	56.9	0.017

PRISMA 2009 Flow Diagram

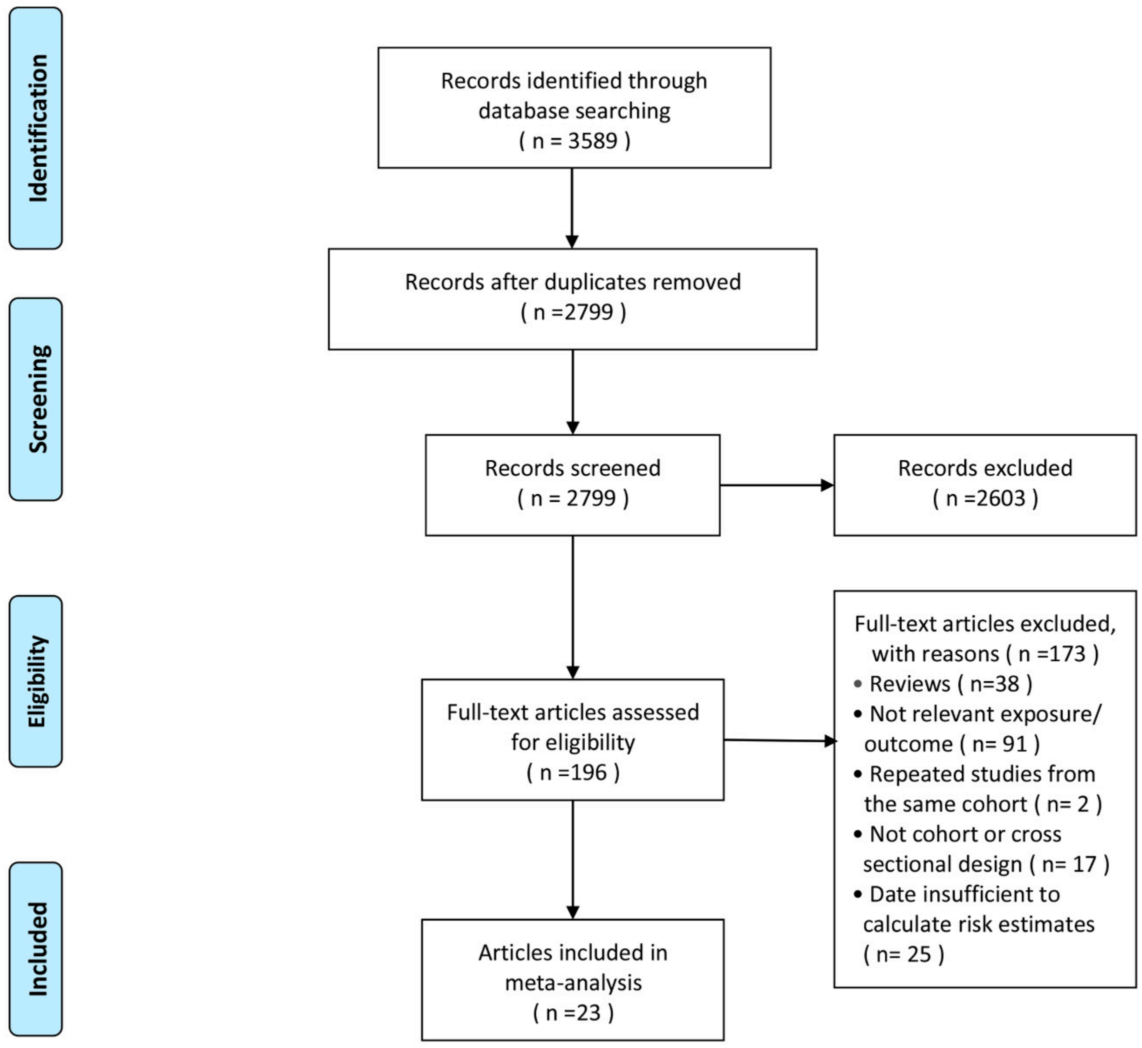

Supplementary Figure 1. flow diagram of the meta-analysis

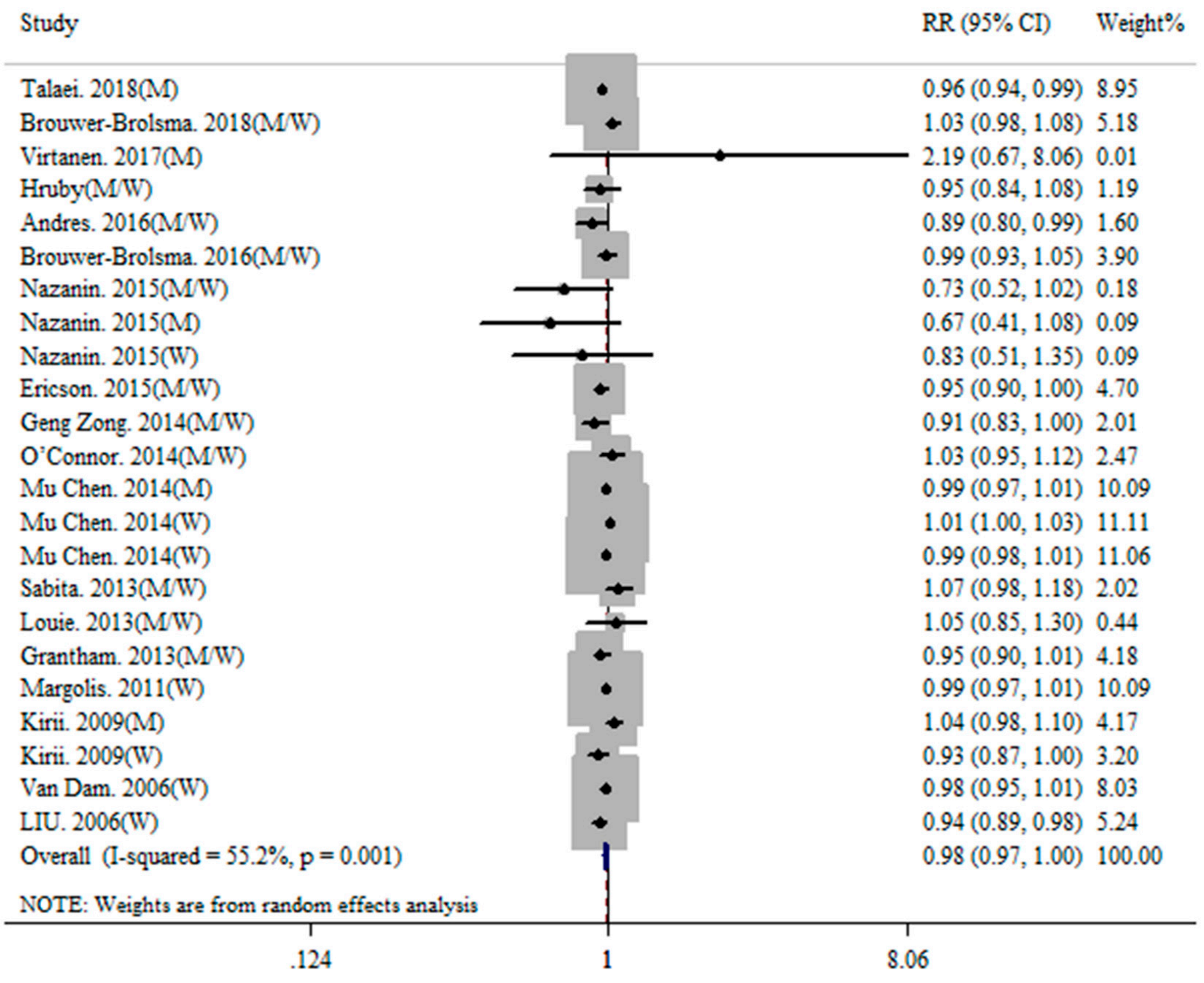

Supplementary Figure 2. Prospective associations of dairy intake with incident type 2 diabetes mellitus for dose-response analysis of the meta-analysis

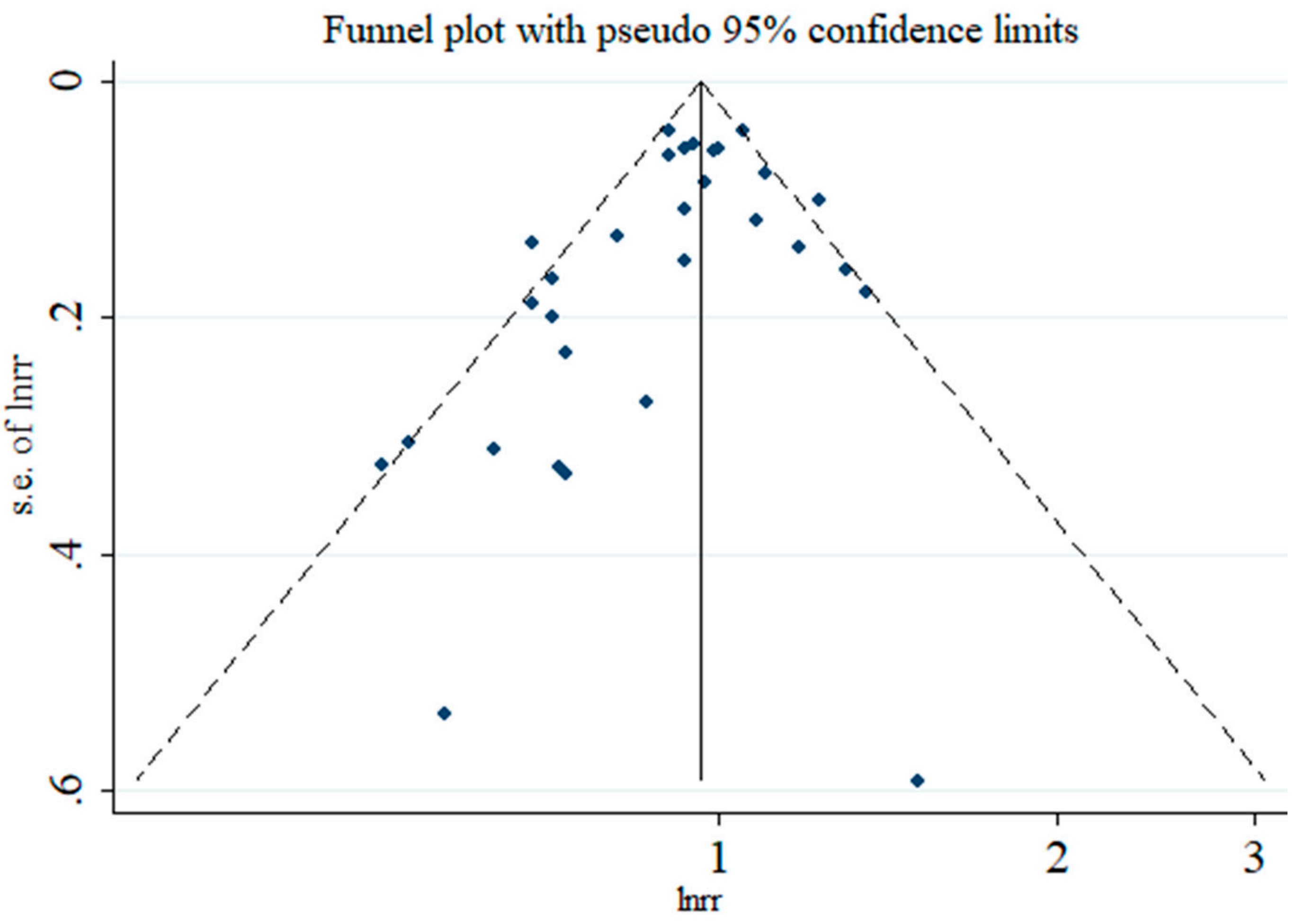

Supplementary Figure 3: Funnel plots of dairy intake-T2D associations (high vs. low meta-analysis) SE = Standard error

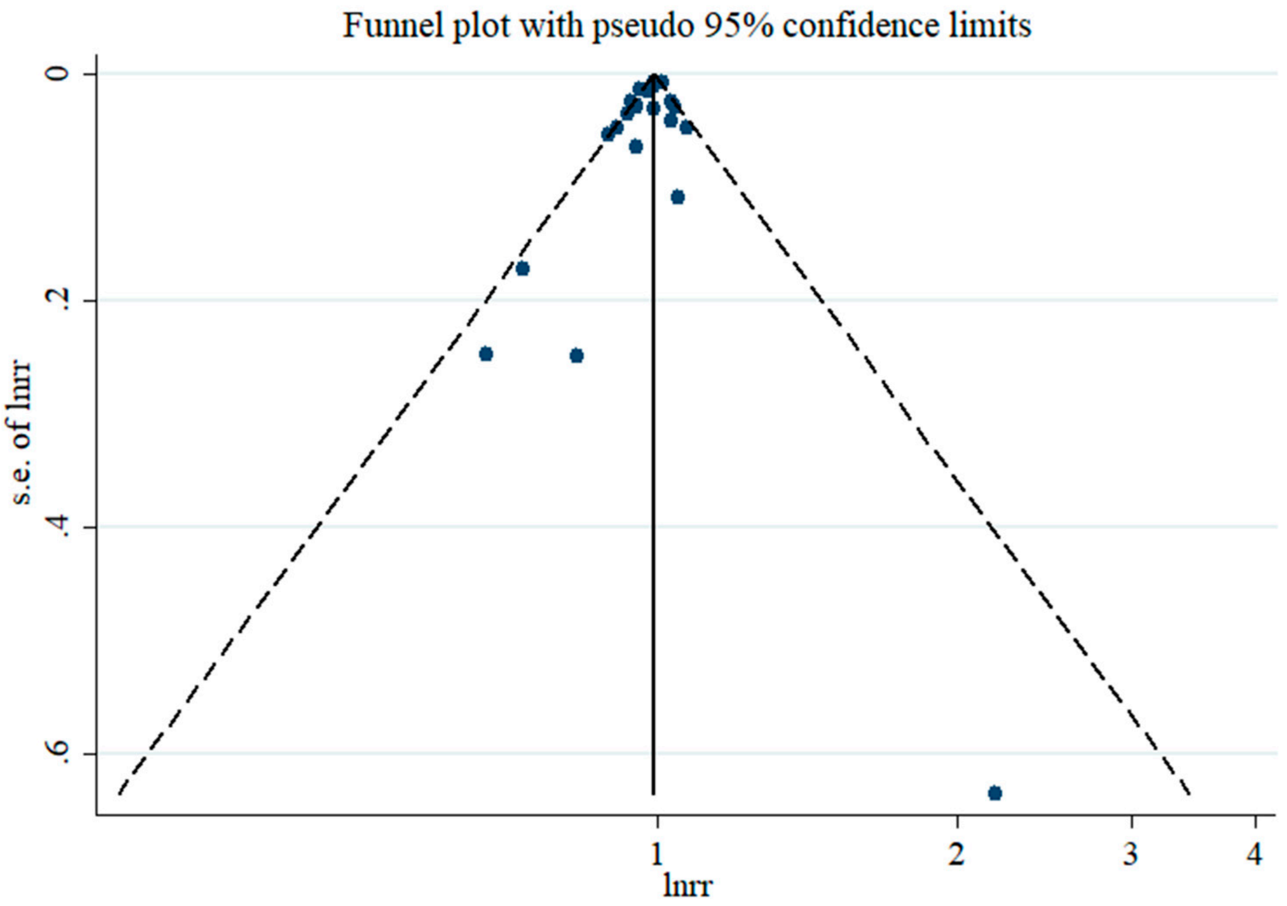

Supplementary Figure 4: Funnel plots of dairy intake-T2D associations (dose-response meta-analysis) SE = Standard error
